# Progress in Skin Diseases

**Published:** 1902-08-02

**Authors:** 


### Progress in Skin Diseases.

**Eczema.**—Bockhart and his colleagues[1] show that the toxins produced in broth by the growth of staphylococci are capable of producing eczema by themselves. On the other hand the poison contained in the bodies of the cocci, called staphyloplasmin only produces pustular diseases. Thus living but inactive cocci may exist in the follicles until some improvement in their food renders them active and virulent. The toxin which they excrete leads to the formation of papules and vesicles. These early vesicles remain sterile unless the cocci migrate into them, which does not necessarily occur. Unna's oration[2] on the history of eczema in the last century in England brings out the curious fact that eczema was unrecognised and undistinguished from other affections a hundred years ago. Even Willan's idea of it was only an acute traumatic vesicular dermatitis of which sunburn is a typical example. Rayer amalgamated with this the group of non-traumatic chronic diseases of the impetigo type. From this combination endless subsequent confusion arose, and Devergie in vain tried to separate the two incompatible groups; even the idea of a dry non-vesicular eczema only originated with M'Call Anderson. The accepted type of eczema with its scales and itching is really that of Rayer but the theories of its causation were endless until Unna himself showed its parasitic origin, giving it thus a clear and simple ætiology. On the other hand Willan's group depends on acute external trauma and was not intended to embrace any of the class of diseases to which the name has been transferred. For a long time the idea that a vesicle was the primary essential lesion in our present eczema prevented men from seeing that seborrhœic eczema was a true form of the disease. This is only one of the hindrances which the confusion of the two conceptions has caused.

**Electro-platers' Dermatitis.**—A. Hall[3] gives an account of the skin troubles caused by "scratch brushing" and polishing. In the former process the articles are cleaned by a revolving brush which is kept wet by sour beer. This splashes over the hands and even the faces of the workers causing an acute dermatitis in many patients. The polishers use a powder called rouge, some kinds of which contain mercury, and a troublesome dermatitis is apt to follow. The writer finds that for dry work no treatment is so good as the application of zinc gelatine and he gives a formula for a cheaper kind which can be made at 8d. a pound and is more adhesive than the ordinary one. His formula is as follows :—

| | oz. |
|---|---|
| Gelatine (Coignets No. 3) ... ... | 16 |
| Calamine (cheap) ... ... ... | 12 |
| Glycerine ... ... ... ... | 20 |
| Water ... ... ... ... ... | 26 |

The gelatine is soaked in 20 oz. of water for 12 hours. The calamine and glycerine are well rubbed up in a mortar, and added to the gelatine and water which are heated, and the mortar washed out with the rest of the water. When cold the jelly is cut into blocks and kept in air-tight boxes. To apply it the jelly is heated over a water bath and painted on in a thin layer. Then a little cotton wool is dabbed over it to form a thin feltwork and allowed to dry. The paint is peeled off at night if necessary and reapplied next day.

**Xerodermia**, which is a mild form of icthyosis, is much improved by small doses of thyroid and by the use of tar baths. Phineas Abraham[4] gives in addition an ointment containing creolin. In place of the French glycerine treatment he sometimes employs baths with 4 drachms of glycerine and 2 of creolin to 6 gallons of water, or a lotion containing the same articles, besides daily inunction with the ointment.

**Dermatitis Gangrenosa.**—T. M. Finny[5] reports a fatal case in a boy two and a half years old, who showed a number of punched out ulcers on the scalp, face, hands, and nates, most of them being of the size of a shilling. There were also sores on the nose and lips, and a measly rash on the legs. No bullæ were present at any time. In spite of nourishing food and creolin and iodoform dressings no healing was observed.

**Alopecia Areata.**—N. Walker[6] found that the affected hairs show a glistening white growth in Sabouraud's medium; in 70 per cent. there was seborrhœa present, but in none was any ringworm to be found. He thinks that the nervous element has been much over-rated. On the other hand, Bayet gives the case of a girl where the hair fell off the front part of the head three weeks after a blow. In this patient there was no sign of seborrhœa. Holzknecht[7] reports a cure in four months by frequent short exposures to the X-rays. The result could not be due to their bactericidal action, for this is very slight. Balzer[8] recommends washing with soap and then with perchloride lotion combined with ether and spirit, and finally friction with lactic acid. Embrick found good results from the application of chysarobin ointment of 10 to 15 per cent. strength daily for a week or ten days; after the inflammation had subsided small areas were touched with pure carbolic acid, and a good growth of hair followed.

**Corns.**—Freeland[9] argues that the removal of pressure and taking away the hypertrophied corneal layers is not enough to effect a lasting cure, for this leaves untouched the stratum mucosum in a state of active growth. He therefore advises complete excision through the subcutaneous tissues. This is done under a local anæsthetic, and the edges are brought together by two or three sutures. He claims that the operation is safe, painless, and gives a permanent cure.

1 Med. Times and Gaz., Dec. 1. 2 Brit. Jour. Dermat., July. 3 Brit. Jour. Dermat., April. 4 Clin. Jour., Dec. 4. 5 Med. Rec., Jan. 11. 6 Treatment, Jan. 7 Brit. Med. Jour., Feb. 22. 8 Loc. cit. 9 Brit. Med. Jour., March 15.

